# *In situ* environment rather than substrate type dictates microbial community structure of biofilms in a cold seep system

**DOI:** 10.1038/srep03587

**Published:** 2014-01-08

**Authors:** On On Lee, Yong Wang, Renmao Tian, Weipeng Zhang, Chun Shum Shek, Salim Bougouffa, Abdulaziz Al-Suwailem, Zenon B. Batang, Wei Xu, Guang Chao Wang, Xixiang Zhang, Feras F. Lafi, Vladmir B. Bajic, Pei-Yuan Qian

**Affiliations:** 1 Division of Life Science, Hong Kong University of Science and Technology, Clear Water Bay, Hong Kong; 2 Sanya Institute of Deep Sea Science and Engineering, Chinese Academy of Sciences, San Ya, Hai Nan, China; 3 Coastal and Marine Resources Core Laboratory, King Abdullah University of Science and Technology, Thuwal, Saudi Arabia; 4 Advanced Nanofabrication, Imaging, and Characterization Core Laboratory, King Abdullah University of Science and Technology, Thuwal, Saudi Arabia; 5 Computational Biosciences Research Center, King Abdullah University of Science and Technology, Thuwal, Saudi Arabia; 6 These authors contribute equally to this work.

## Abstract

Using microscopic and molecular techniques combined with computational analysis, this study examined the structure and composition of microbial communities in biofilms that formed on different artificial substrates in a brine pool and on a seep vent of a cold seep in the Red Sea to test our hypothesis that initiation of the biofilm formation and spreading mode of microbial structures differs between the cold seep and the other aquatic environments. Biofilms on different substrates at two deployment sites differed morphologically, with the vent biofilms having higher microbial abundance and better structural features than the pool biofilms. Microbes in the pool biofilms were more taxonomically diverse and mainly composed of various sulfate-reducing bacteria whereas the vent biofilms were exclusively dominated by sulfur-oxidizing Thiomicrospira. These results suggest that the redox environments at the deployment sites might have exerted a strong selection on microbes in the biofilms at two sites whereas the types of substrates had limited effects on the biofilm development.

In the marine environment, surfaces of any submerged materials tend to quickly adsorb organic molecules that form into a surface organic layer. Such an organic layer often favors the attachment and eventual colonization by microorganisms, including bacteria, archaea, diatoms, protozoa, and fungi, which become enmeshed in a matrix of extracellular polymeric substances (EPS) to form what is collectively known as ‘biofilm’^1^. Biofilm architecture provides an effective strategy for microorganisms to survive in unfavorable environments and to colonize new niches^2^. Biofilm formation involves initial cell-surface interactions, cell attachment, maturation, and finally dispersion^3^. At the initial stage when microorganisms approach a surface, the physical properties of the surface, such as surface energy, hydrophobicity, and roughness, play important roles in determining which microorganisms can successfully attach^4^,^5^. Subsequently, EPS production by microbes leads to their establishment on the surface, followed by alterations of the microbial physiological responses to conditions in their specific niches and cell-cell interaction^3^. These processes can be affected by environmental or external signals that trigger corresponding genetic and regulatory circuits during biofilm development^6^ and lead to substantial differences in biofilm structure and composition. However, the development models of biofilms were largely based on experimental results in laboratories, and only few studies were conducted in complex natural environments. Considering the importance of studies on biofilms in different ecosystems, a growing number of researches have reported microbial structures of natural biofilms and ecological factors determining their development. For example, intertidal and subtidal biofilms were diversified by metal ion and oxidative stresses^7^, and biofilms from different streams were analyzed to support the species sorting theory on microbes assembly from different sources^8^.

So far, there has been only one study that reported biofilm formation on artificial substrata in water column from surface water to deep-sea waters^9^ but there has been no study to reveal formation mechanisms of biofilms in deep-sea extreme environments, such as cold seeps and brine pools. Cold seeps are formed by subsurface fluid expulsions to the seabed, due to differential pressure gradients and tectonic activity at both active and passive continental margins^10^,^11^. They normally do not show any marked temperature anomaly. The vertically migrating subsurface fluids at cold seep areas are often rich in hydrogen sulfide, methane, and other hydrocarbons^11^. In some cold seep settings, hypersaline water (brine) flows out of the seabed and eventually forms a pool if the bottom topography allows for localized containment of the brine fluids. Many previous studies have reported the prevalence of thiotrophic (sulfur-oxidizing), sulfide-oxidizing, sulfate-reducing and/or methanotrophic (methane-oxidizing) bacteria and archaea in microbial mats^12^,^13^, sediments^14^,^15^,^16^, and as symbionts^17^,^18^,^19^,^20^,^21^ in cold seeps. Microbial community diversity and dynamics in biofilms on artificial substrata in such environments have not been examined. Microbial mats therein are formed on sediments enriched with organic matter whereas biofilm grown on a solid surface was initiated by the accumulation of organics. Therefore, availability of the sticky matter in the cold seeps is likely a bottleneck for the formation of biofilms. We hypothesized herein that initiation of the biofilm formation and spreading mode of microbial structures differ between the cold seeps and the other aquatic environments. In addition, it is also unclear to what extent the types of artificial substrata might affect the microbial assembly on the biofilms. In this study, we deployed artificial substrates of different materials at a newly-discovered cold brine seep system at about 840–850 m depth on the continental margin of the central Red Sea, the Thuwal Seeps^22^, to examine the biofilm development in its brine pool and seeping vent and also on different types of substrates, using 16S rDNA tag pyrosequencing, clone library, and scanning electron microscopy.

## Results

### Environmental conditions and characteristics of biofilms

The seeping water from the seep vent was characterized by high salinity, sulfate, nitrate, silica and manganese contents (Table 1). The cell density in the seeping water was approximately 96 × 10^3^ cells/mL. The two biofilm development platforms were deployed within the pool and at the seep vent of Thuwal Seeps at 50 m apart. The temperature and salinity recorded near the deployment site within the pool were 21.3°C and 71.2‰ while those at the fresh brine outflow from the seep vent were 29.5°C and 124.6‰. From the surface to the bottom of the brine pool, dissolved oxygen (DO) concentration decreased from about 24% to nearly 0.5%; DO at the deployment site in the brine pool was estimated to be < 5%. The duration time of the biofilm development in the seeping water (hereafter SV-biofilms) was 100 hours, which was longer than that of 72 hours for the biofilms developed in the brine pool (hereafter BP-biofilms). SV-biofilms were considerably higher in microbial cell density and biomass (volume) than BP-biofilms as shown by the measurements on polyvinyl chloride (PVC) plate (Table 2). The effect of substratum position on the cell densities was displayed by BP-biofilms. The BP-biofilms from the outer surfaces of the platform had apparently higher cell densities than those from the inner. Substratum materials also affected the development of the BP-biofilms, as biomass (volume) on PVC was higher than those on PS plates (Table 2). However, the coverage of the biofilms was relatively lower on PVC than on PS plates. This was stemmed from the presence of agglomerated masses or clumps on the BP-biofilms developed on the outer PVC plates, which were not frequently observed on PS plates. As viewed under the scanning electron microscope (SEM), such clumps were common in BP-biofilms on PVC but not on PS plates (Fig. S1). Similar phenomenon was observed in SV-biofilms developed on PVC plates; clumps of up to 83 μm high and 62 μm wide were more frequently encountered. A 3-way ANOVA detected significant effects of deployment site on cell density (F = 14.59, p-value = 0.002), mean thickness (F = 6.481, p-value = 0.026), and coverage (F = 5.406, p-value = 0.038), and of plate position on mean thickness (F = 5.501, p-value = 0.037), but surprisingly no significant impact of the type of substrates on any of the parameters measured. In addition, no interactions between site, material and plate position on the parameters were detected.

### Diversity and composition of microbial communities revealed by pyrosequencing

Pyrosequencing produced nearly 150,000 raw reads per replicate mixture. Quality filtering removed 20–25% of the raw reads. Rarefaction analysis based on operational taxonomic units (OTUs) at 3% of dissimilarity showed that the rarefaction curves were approaching plateaus, particularly for the SV-biofilms, suggesting that there were sufficient sequences to represent the whole microbial communities. The number of OTUs and Shannon index of the microbial communities are shown in Tables 3 and S1. The number of OTUs obtained from the biofilm samples ranged from 37 to 410 while that from free-living microbes in the seeping water (SW) was 401. Generally, the microbial diversity, in terms of species richness and Shannon index, for the SV-biofilms was far lower than that for the BP-biofilms, the latter being more comparable to that of the free-living community (Table 3). At each location, no significant trend was detected in microbial diversity of the biofilms, regardless of material type and plate position on the platform.

**Figure 1:**
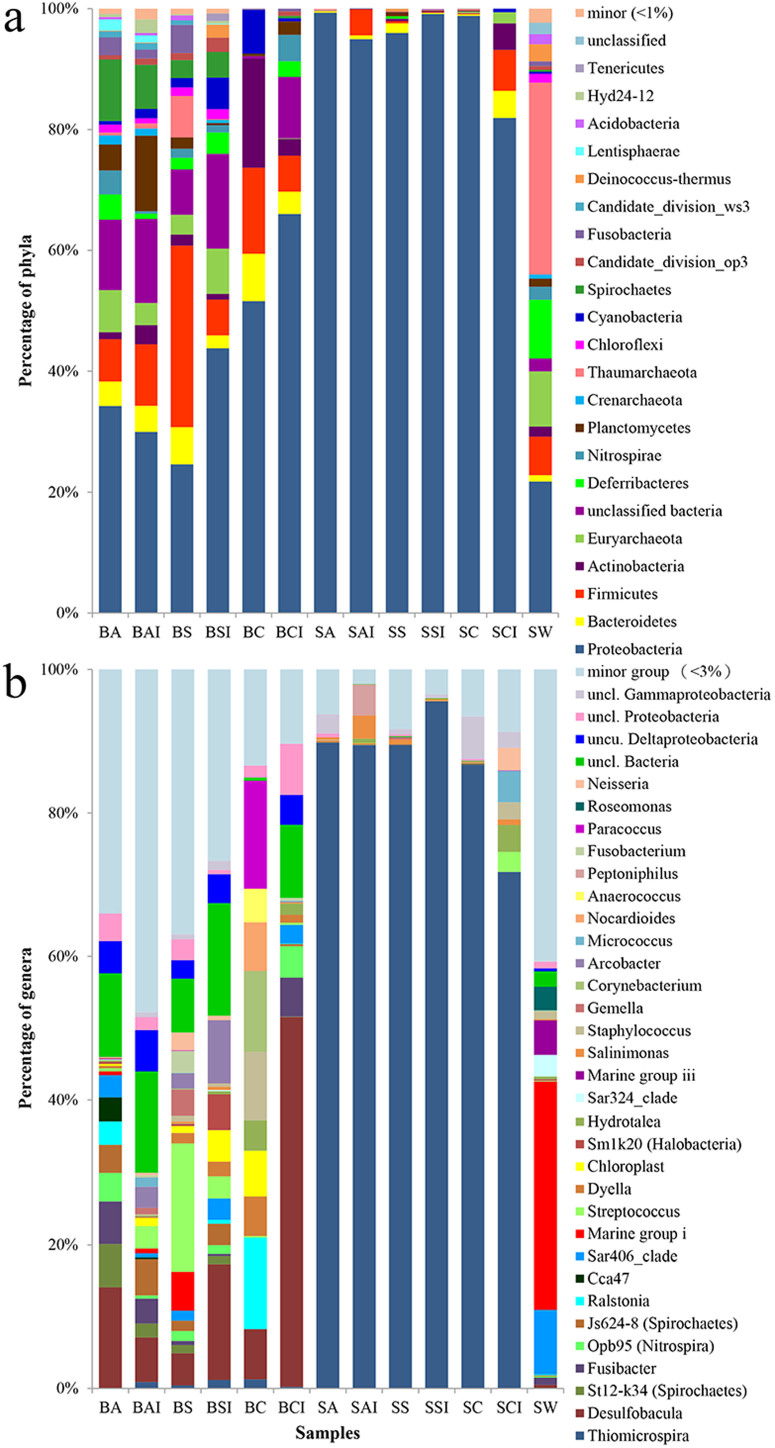
Percentage of microbial taxa classified based on 16S rRNA gene sequences. Biofilms were developed on different substrate materials within the brine pool (BP-biofilms) and on a seeping vent (SV-biofilms). The 16S rRNA amplicons of the seeping water, BP-biofilms and SV-biofilms were pyrosequenced and classified by comparing with the SILVA database at (a) phylum and (b) genus levels. Minor group represents the sum of all phyla or genera with a proportion of less than 1% and 3%, respectively, for all 13 samples. Refer to Table 3 for sample ID.

A total of 114,203 out of 119,439 qualified reads (95.6%) were assigned to known phyla. For the replicates of BP-biofilms, the microbial communities differed remarkably among the samples (Figs. 1 and S2). In contrast, microbial communities in the replicates of SV-biofilms were consistent and all were overwhelmed by the phylum Proteobacteria. Mann-Whitney tests showed that the BP-biofilms developed on aluminum (inner) and PVC were significantly different (*p* < 0.02); the SV-biofilms developed on aluminum (outer), PS (inner) and PVC (inner) substrata were also significantly different in terms of microbial composition (*p* < 0.05). However, the significant differences in SV-biofilms were ascribed to unevenness of the minority species. Overall, 39 phyla were detected from the biofilm and seeping water samples, with 71.4% of the qualified reads belonging to Proteobacteria (Table S2). About 18% of reads were sorted into other 7 phyla, including Firmicutes, Thaumarchaeota, Euryarchaeota, Bacteroidetes, Spirochaetes, Planctomycetes, and Deferribacteres. The remaining sequences were assigned to additional 31 phyla or unassigned. In the SV-biofilms, Proteobacteria accounted for 81.9–99.3%, but its percentage dropped to 24.6–66.0% and 21.8% in BP-biofilms and SW sample, respectively (Fig. 1a, Table S2). BP-biofilms also harbored other dominant bacterial phyla, including Euryarchaeota, Actinobacteria, Spirochaetes, Firmicutes, and Bacteriodetes. However, the dominant position of these phyla was not consistent between the replicates of BP-biofilms, as indicated by the results of Mann-Whitney tests (Figs. 1 and S2). Instead, the free-living community in SW was mainly composed of Thaumarchaeota, Euryarchaeota, and Deferribacteres.

For the SV-biofilms, the number of genera with relative abundance higher than 3% in the SV-biofilms ranged from 9 to 12, except for the one on PVC surface with 21 (Table S3). Gammaproteobacterial *Thiomicrospira* dominated exclusively and accounted for 71.8–95.6% of the qualified reads per sample (Fig. 1b). Reads assigned to *Thiomicrospira* comprised of three OTUs that shared 96% similarity with *T. frisia* strain JB-A2. In contrast, more genera (up to 174 genera) were observed in the BP-biofilms and SW sample, although many of these genera were in low abundance. The relative abundance of the different genera in the BP-biofilms varied substantially, with a ubiquitous presence of *Desulfobacula* that accounted for 4.5–51.3% of the reads (Fig. 1b). SW was dominated by the Marine Group I archaea, although there were also appreciable representations by the Marine Group III archaea and the SAR406 clade of the bacterial phylum Deferribacteres.

### Similarity and ordination of biofilm microbial communities

**Figure 2:**
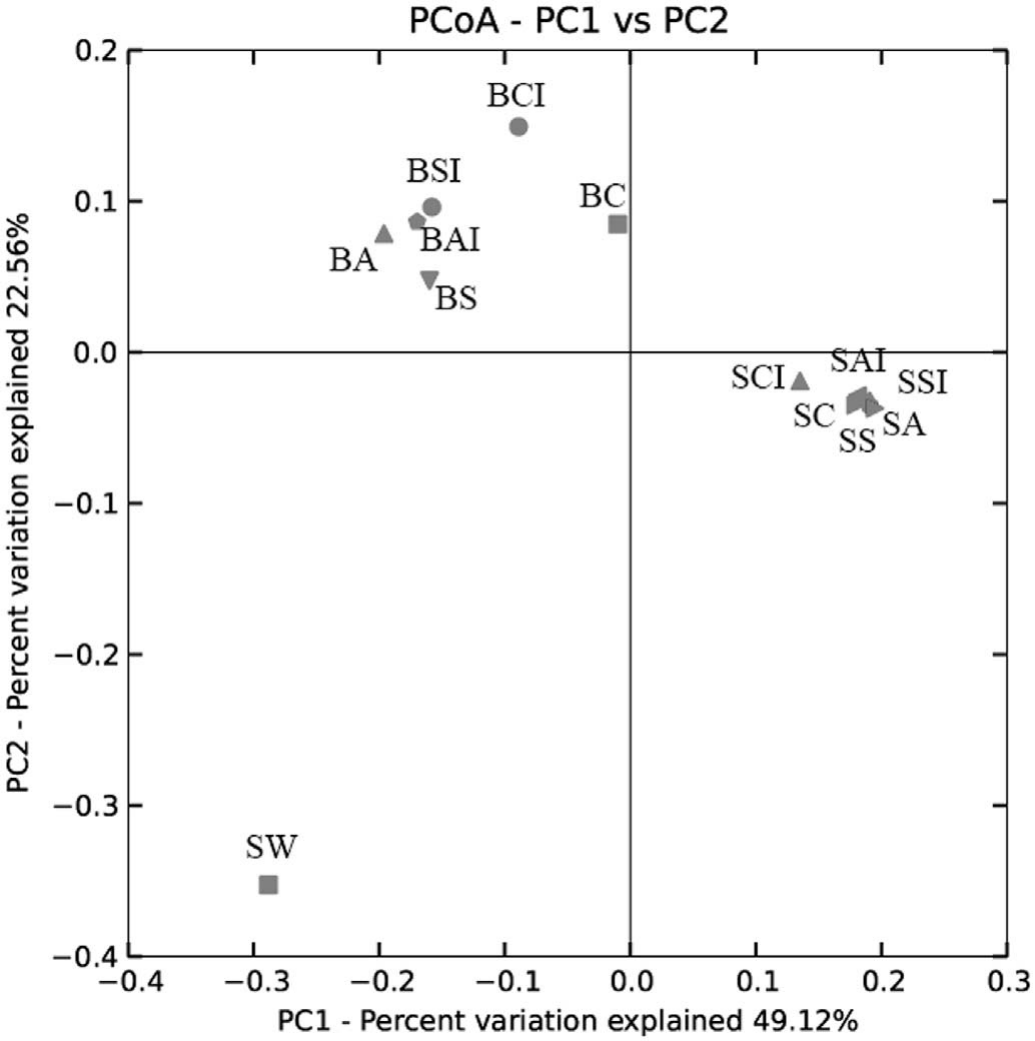
Principal coordinates analysis (PCoA) of microbial communities of seeping water and biofilms. The microbial communities were revealed based on 16S rRNA gene pyrosequencing. PCoA plot of PC1 and PC2 is shown. Sample IDs are described in Table 3.

Clustering and ordination analysis (Figs. 2 and S3) showed substantial differences in microbial communities between biofilms and seeping water samples. The first principal coordinate (PC1, with an explained variance of 49.1%) distinguished the microbial communities of BP-biofilms and SW from the SV-biofilms, while PC2 (22.6%) largely separated the SW community from the biofilm communities (Fig. 2). Hence, results of the PCoA agreed well with the distinctive clustering of the microbial communities by sample site with high bootstrap values in the Jackknifed-UPGMA tree (Fig. S3). Higher variations in microbial communities were observed among BP-biofilms than among SV-biofilms, as evident by close clustering with short distances between the SV-biofilm samples.

**Figure 3:**
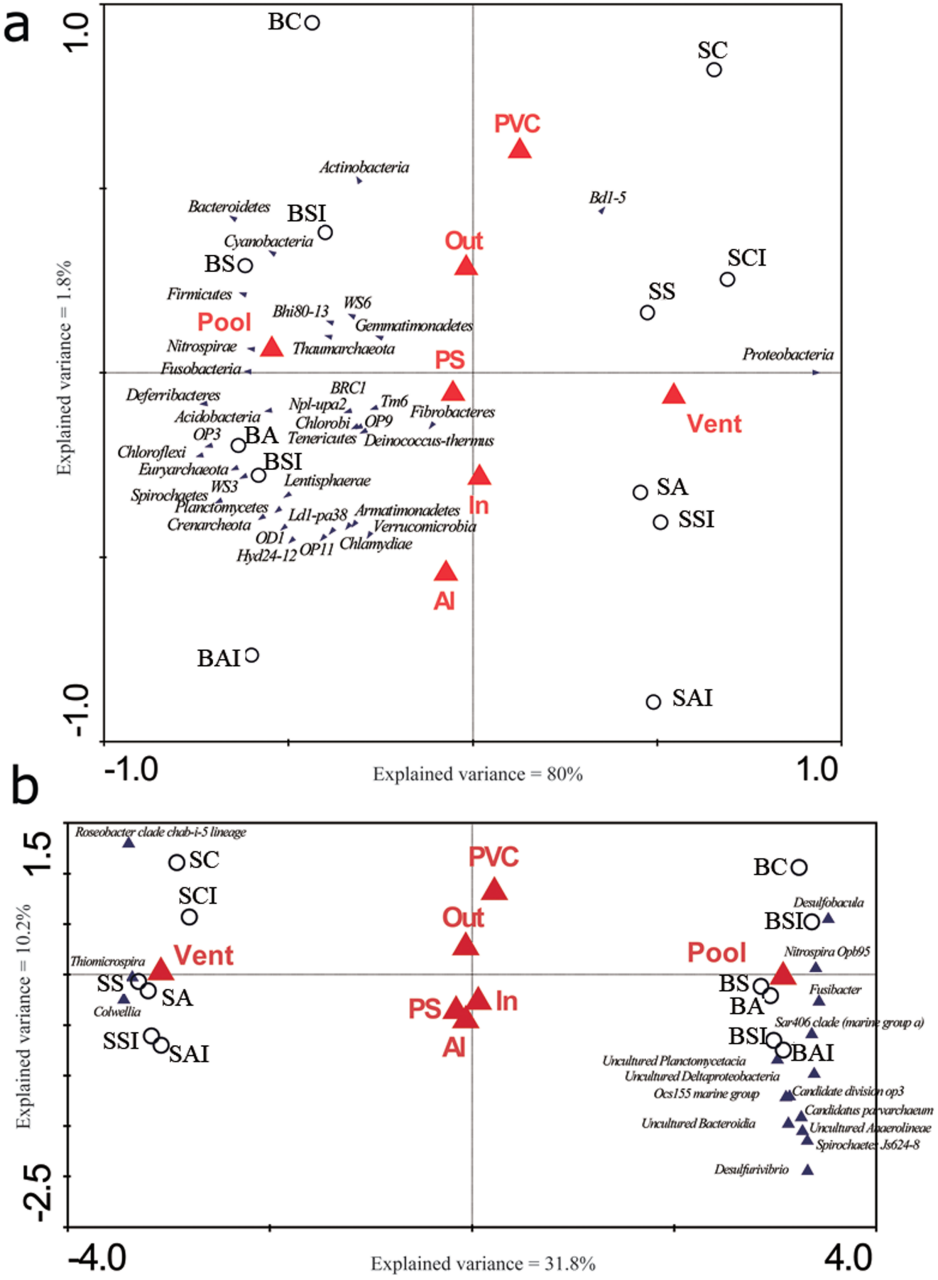
Ordination analysis (CANOCO) of biofilm microbial abundance in relation to site, substrate material and plate position. Percentage of 16S rRNA pyrosequencing reads assigned to (a) phylum and (b) genus levels was used as the ‘species data’ for RDA and CCA analyses, respectively. Correlations between environmental variables (marked by red triangles; site: vent and pool; material type: Al, PVC and PS; and orientation: in and out) and the first two canonical axes are represented by the length and angle of the blue triangles (environmental factor vectors). Biofilm samples were denoted with open circles. Forward selection with Monte Carlo permutation tests was applied to build the parsimonious model, which identified site as the major influential factor significantly contributing to the variations in the biofilm microbial communities. At the genus level, the lower axis minimum fit was set to the variance explained by the first axis to identify genera which were affected most severely in the model.

The relationships between microbial community composition and three factors (namely site, substrate material, and plate position on the platforms) were further examined by ordination methods. Results of CANOCO analysis showed the type of substrate and plate position have an insignificant effect on microbial community composition, regardless of taxonomic level (*p* > 0.820, Table 4). In contrast, the site and the combination of all three factors were better determinants of the variations in microbial community composition, as indicated by their high explained variances (sums of all canonical eigenvalues in Table 4) at both taxonomic levels. In both RDA and CCA triplots (Fig. 3), the first axis largely separated the BP-biofilms from the SV-biofilms, while the second axis had relatively minor effects but marginally discriminated the microbial communities between Al and PVC plates (Fig. 3). The abundance of Proteobacteria showed a strong positive correlation with the SV-biofilms, whereas strong positive correlations were observed for Nitrospirae, Fucobacteria, and Thaumarcheota with the BP-biofilms (Fig. 3a). Specifically, *Thiomicrospira* and *Colwellia* abound in the vent biofilms whereas *Desulfobacula*, *Nitrospira* Opb95, *Fusibacter* and the SAR406 clade, *Spirochaetes* Js624-8, and *Desulfurivibrio* in the pool biofilms (Fig. 3b). Surprisingly, the microbial community structure at both taxonomic levels appeared to be largely unaffected by plate position.

### Presence of bacteria involved in sulfur cycling

**Figure 4:**
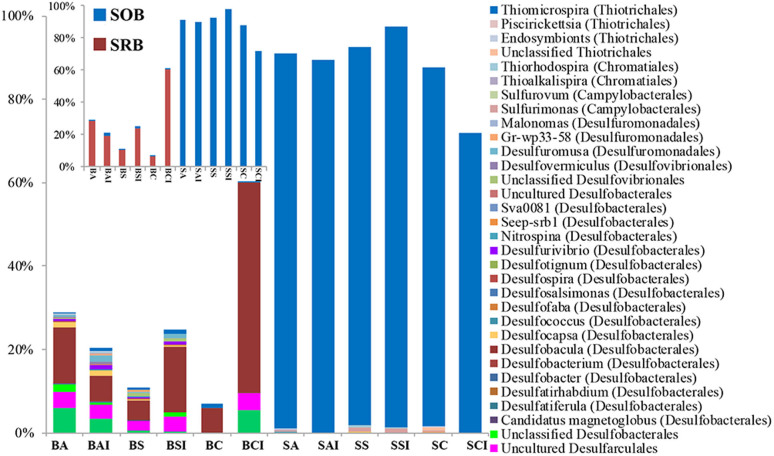
Percentage of 16S rRNA gene pyrosequencing reads assigned to different groups of sulfate-reducing (SRB) and sulfur-oxidizing (SOB) bacteria in different biofilms. Inset shows the abundance of total SRB and SOB. Taxonomic classification was based on comparison of sequences within the SILVA database.

With relative abundance of up to 60%, sulfate-reducing bacteria (SRB) proliferated in the BP-biofilms. The identified SRB taxa included *Desulfarculales*, *Fusibacter*, *Desulfatirhabdium*, *Desulfobacterium*, *Desulfobacula*, *Desulfocapsa*, *Desulfofaba*, *Desulfospira*, *Desulfotignum*, *Desulfurivibrio*, *Desulfovibrionales*, *Desulfuromusa*, *Desulfuromonadales*, *Nitrospina*, Candidatus *Magnetoglobus*, Gr-wp33–58, and Seep-srb1 (Figs. 4 and S4). These SRB were affiliated with three phyla, out of 8 major known phyla. In contrast, SRB belonged to only 3 to 5 genera with overall relative abundance below 0.3% in the SV-biofilms that were dominated by sulfur-oxidizing bacteria (SOB), mainly *Thiomicrospira* (up to 72–96%). Other known or suspected SOB genera in the SV-biofilms included *Sulfurimonas*, *Sulfurovum*, *Thioalkalispira*, *Thiorhodospira*, *Endosymbionts*, and *Piscirickettsia*. In contrast, the relative abundance of SOB in the BP-biofilms was between 0.4% and 1.6% (Fig. 4).

**Figure 5:**
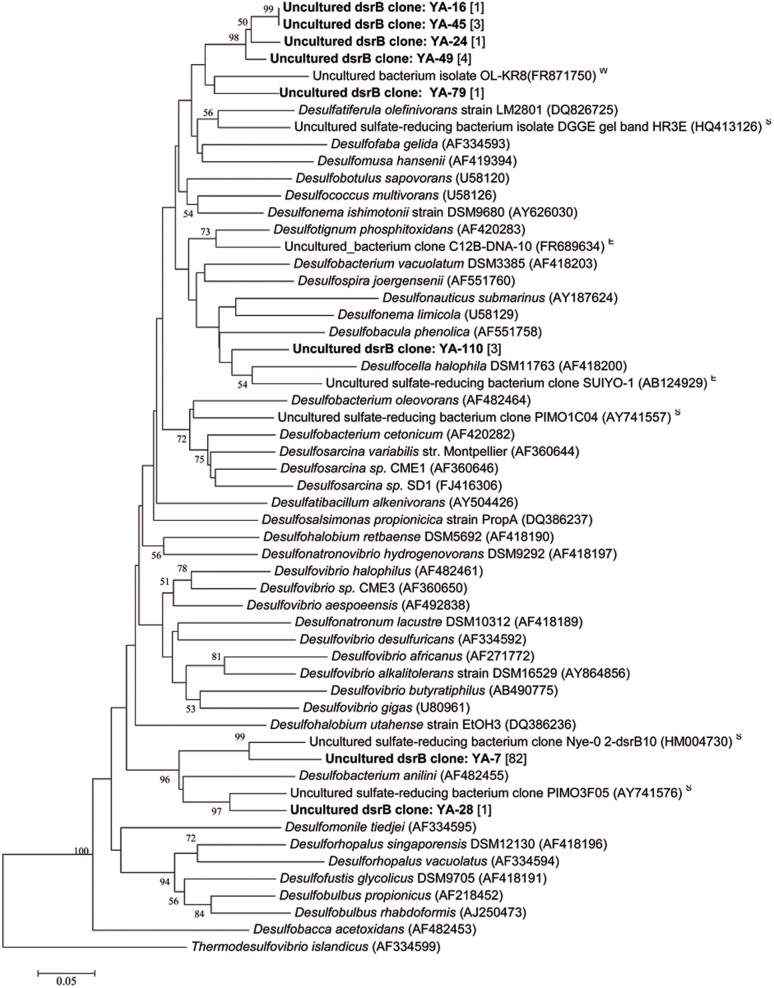
Phylogenetic trees based on sequences of *dsrB* gene obtained from the BP-biofilm and SW samples, respectively. The trees were constructed using Neighbor-Joining method. Bootstrap values of > 50% based on 500 resamplings are indicated by numbers at nodes. Each clone sequence (OTU) is represented by sample ID followed by clone number in bold. Number in bracket indicates number of clones assigned to the same OTU. Reference sequence is represented by strain name followed by accession number in parenthesis. The isolation source of the reference clone sequence is indicated by a superscript of ‘W’ for water, ‘S’ for sediment, and ‘E’ for environmental sample.

Since SRB dominated the BP-biofilms, we constructed a clone library for functional gene *dsrB* to verify their function in sulfur reduction. The BP-biofilm on aluminum plate was selected for library construction, considering its relatively high microbial diversity based on the 16S rDNA pyrosequencing data. There were 96 *dsrB* positive clones and their sequences were grouped into only 8 OTUs, which were lower than the expected. As indicated in Figure 4, the analysis of 16S rDNA amplicon reads revealed 17 *dsrB* OTUs. Among the 8 OTUs, the most dominant OTU (YA-7), with 82 sequences assigned, shared a high similarity with an uncultured SRB from the Nyegga pockmark sediment in the Norwegian Sea; the second most dominant OTU (YA-110) was closely related to *Desulfobacula* *phenolica* and *Desulfocella* *halophila* (Fig. 5); other 5 OTUs (YA-16, YA-45, YA-24, YA-49 and YA-79) clustered with a clone sequence from saline groundwater at 500 m deep at Finland; and the last OTU (YA-28) was similar to a clone from the sediment of a salt marsh in Plum Island, New England and the SRB *Desulfobacterium aniline*.

## Discussion

The free-living (planktonic) microbial community is regarded as the source of colonizers of submerged surfaces in the marine environment. At Thuwal Seeps, the present results showed a lower microbial diversity in biofilms than in free-living community, supporting the sorting effect of substrata on the free-living microbes in the process of biofilm formation. Moreover, distinct differences in taxonomic compositions were displayed between BP-biofilms and SV-biofilms. Sulfate-reducing Deltaproteobacteria, especially *Desulfobacula*, were clearly the dominant microbes in the BP-biofilms, regardless of the type of substrates, but the same bacteria were not highly represented in the SV-biofilms. Conversely, the thaumarchaeota MGI, which dominated the planktonic community, was barely detectable in the BP-biofilms. The drastic differences in dominant bacterial species between BP-biofilms and SV-biofilms probably resulted from the *in situ* conditions. The relatively high concentration of sulfate, up to 2.6 g/L, in the brine pool may promote proliferation of SRB in the BP-biofilms. In addition, the attached mode of life as biofilms may afford greater advantages than a free-living mode for many SRB, due to the higher efficiency in intermediate transfer by molecular diffusion^22^ and higher survivor rate^23^. In fact, previous studies show that thiotrophic, sulfate-reducing, and methanotrophic bacteria and archaea dominate biofilm communities in cold seep microbial mats^12^,^13^, sediments^13^,^14^,^15^, and also macrofauna through symbiosis, such as in bivalves^17^,^19^,^20^, sponges^21^, and tubeworms^24^. The current study showed for the first time that SRB dominated the BP-biofilms on the solid surfaces.

In this study, the biofilm microbial community thriving in the seep vent at Thuwal Seeps was almost exclusively made up by *Thiomicrospira*. This genus was established in 1972 when an obligate chemolithotroph capable of sulfur oxidization was isolated from intertidal mud^25^. At present, this genus contains 10 validly published species that were isolated from saline, mostly marine, habitats including coastal mud flats, sediments, and hydrothermal vents^26^. Members of this genus are considered as chemolithotrophs that oxidize reduced sulfur compounds, such as sulfide, thiosulfate and elemental sulfur, to generate energy and fix carbon dioxide by genes encoded for a key enzyme, ribulose-1,5-bisphosphate carboxylase/oxygenase (RubisCO), involved in the Calvin–Benson–Bassham cycle^26^. *Thiomicrospira* oxidizes thiosulfate via the S_4_I pathway that is different from the PSO pathway involving the *SoxB* gene in most SOB^27^. This may also explain why we were unable to PCR-amplified *soxB* gene from the SV-biofilm samples for clone library construction (unpublished data). Since the Thuwal cold seeps discharged the solutions with a high concentration of H_2_S^28^, it is possible for the chemolithotrophic microbes near the vents to proliferate. The prevalence of *Thiomicrospira* in the SV-biofilms provides strong evidence of the strong bacteria-mediated sulfur oxidization at the seepage site. Oxidation of the hydrogen sulfide could result in a high concentration of biogenic and abiogenic sulfate in the seeping water before influx into the brine pool. Since the brine pool water contains an extremely low concentration of oxygen, sulfate serves as the major oxygen donor and thus, the SRB shall prevail in the brine pool. Indeed, our results showed that the bacterial groups responsible for different steps of sulfur cycling in this cold seepage system proliferated in BP-biofilms and SV-biofilms, separately. In summary, the redox conditions in the brine pool and seeping vent differentiated the primary producers, which then led to the distinct microbial communities in the biofilms at different sites.

The rate of development, microbial composition, and quality of biofilms might significantly differ between substrate materials as shown in previous studies^5^,^29^,^30^. However, results of the present study did not support a definitive impact of substrate material on resulting biofilms at both deployment sites. So far, only a few studies have been devoted to biofilm development on artificial surfaces in deep-sea environment. Nevertheless, a recent study reported that substrate types and orientation were less important than depth in shaping the microbial composition of deep-sea biofilms at 1,500–4,500 m depth in the eastern Mediterranean Sea^9^. Our results also support the importance of environments, rather than substrata, in shaping the microbial structures of biofilms. Although we saw a drastic impact of deployment sites on microbial community structure of the biofilms, due to constrains of experimental design, exposure duration, and type of substrates used in this study, it is premature to generalize the effects of substrates on biofilm development in the cold seep environment.

This study showed drastic differences in morphological features and taxonomic composition of microbial communities between the pool and vent biofilms. The substantially different environments may have shaped microbial community structures^31^,^32^. Other driving forces, for instance soak-time or flow dynamic, may also affect the success and rate of microbial colonization on surfaces and subsequent biofilm composition^29^,^33^. On the other hand, the results of this study do provide first-hand information of the potential impacts of microbial colonization on microfouling and biocorrosion of submerged structures and equipment in similar deep-sea and extreme environments. To gain a much better understanding of the type of substrates and environmental conditions on biofilm development and to verify our preliminary findings, improved experimental design and longer deployment times are required. The ultimate outcome of such studies will help us select suitable materials for deep-sea research facilities.

## Methods

### Study area and experimental setup

Thuwal Seeps, as first described during its discovery in May 2010^28^, had two separate seepage outlets that actively discharge brine fluids into an adjacent brine pool. These seepage outlets occur on a raised embankment along the base of a steep wall rock on the eastern side of the pool. However, when Thuwal Seeps was revisited in November 2011 as part of the KAUST Red Sea Expedition 2011 and during which the present experiment was conducted, four new seepage outlets (or seep vents) were found, thus indicating a highly dynamic subsurface fluid expulsions at Thuwal Seeps. Brine outflows from the seep vents, which are densely lined with white bacterial mats, were clearly evident by direct video observations during the study.

For the present biofilm experiment, PS and PVC plates were glued to both the inner and outer sides of two mounting platforms made of aluminum sheets; one platform was deployed on one of the newly found seepage outlets on the eastern bank of the pool (22.2836 N, 38.8983 E) by a remotely operated vehicle (ROV *Max Rover*, DSSI, USA) while the other was placed in the brine pool (22.2834 N, 38.8979 E), with two sites being about 50 m apart. The platform set in the pool had a pyramidal shape, with a top open chimney, while that on the seep vent assumed an inverted wedge, with a top roof and an outflow opening on one side. The latter was so designed to allow for stable placement on top of the seep outlet on the high-sloping embankment, such that the brine discharges are entirely enclosed and the side vent ensuring unimpeded brine outflow to the nearby pool. The ROV, equipped with built-in CCD color video system and 5-function manipulator arm, was remotely controlled onboard *R/V Aegaeo* through an umbilical cable. Due to operational and technical constrains of ROV diving, the two platforms were deployed on different days but retrieved on the same day (the last day of the cruise leg), resulting in different duration of biofilm development; the durations for biofilm development were 72 h and 100 h in the brine pool and on the seep vent, respectively. After hauling in each platform on the ship deck, biofilms on the same surface areas (about 30 cm^2^ each; n = 2) of each substrate type, including PS, PVC and Al, were immediately harvested with sterile cotton tips. The cotton tips were then frozen in 0.8 mL of DNA extraction buffer (100 mM of Tris-HCl, 100 mM of Na_2_-EDTA, 100 mM of Na_2_HPO_4_, 1.5 M of NaCl, 1% CTAB; at pH 8). Seeping water was collected simultaneously from the vent using a 5-L Niskin bottle rigged to the front of ROV for comparison with the biofilm samples. Environmental parameters including salinity, temperature, pH, DO and methane concentrations in brine were measured by a CTD unit mounted on the ROV. Other environmental parameters including sulfate, manganese, iron, copper, silica, nitrate, nitrite, and phosphorus were determined on-board by a portable data logging colorimeter (DR/890, HACH, USA) according to the manufacturer's protocol. A small volume of the water was fixed in 4% formaldehyde for the determination of microbial cell density. The rest of the sample was immediately filtered with 1.6 μm glass fiber membrane (dia. 125 mm, Whatman) to first remove suspended particles and then with 0.22 μm polycarbonate membrane (dia. 47 mm, Millipore) to retain the microbial cells. The polycarbonate membrane was frozen in 0.8 mL DNA extraction buffer. In addition, some biofilms were fixed in 4% formaldehyde for microscopy analysis.

### Microscopy analysis of biofilms

The morphological properties of the biofilms on the PVC and PS plates were examined with confocal laser scanning microscope (CLSM) and SEM but it was then impossible to conduct similar analysis on the biofilms developed on Al plates. Biofilms for CLSM were first stained with fluorescein isothiocyanate-conjugated concanavalin A (Sigma, United States) at 1 mg/mL and then visualized under a CLSM (LSM7 DUO 710, Carl Zeiss, United States) at 40× magnification. For each biofilm, 3 replicates were used for taking Z-stack images. The stacked CLSM images were then analyzed for microbial cell density, biovolume, mean thickness, roughness, and coverage using the image quantification tool PHLIP^34^. For SEM, biofilm samples were dehydrated in serial concentrations of ethanol (30, 50, 70, 85, 95, and 100%; 15 min each), critical-point dried (CPD-2, Pelco, United States), and sputter coated with gold (Scancoat Six, Edwards, United Kingdom) prior to examination under a SEM (JSM-6700F, JEOL, Japan).

### Pyrosequencing of barcoded 16S rRNA gene amplicons

Total genomic DNA was extracted from the biofilm and seeping water samples following the modified SDS-based method^35^ and purified by MoBio Soil DNA Isolation Kit (MoBio Laboratories, USA). DNA quality and quantity were checked with a NanoDrop spectrophotometer (ND-1000, NanoDrop, USA) and gel electrophoresis. Purified DNA samples from two replicates were kept at −20°C for future use.

The hypervariable regions V6 to V9 of the 16S rRNA genes from different biofilm and seeping water samples with replicates were PCR-amplified using the universal forward primer U905F (5′-TGAAACTYAAAGGAATTG-3′) and the reverse primer U1492R (5′-GGTTACCTTGTTACGACTT-3′)^36^ added with unique 8-nucleotide barcodes (Table 2) designed with Barcrawl^37^. A 25-μL reaction contained 1 U of *Pfu* Turbo DNA polymerase (Stratagene, CA, USA), 1 × *Pfu* reaction buffer, 0.1 μM of each barcoded primer, 0.2 mM of dNTPs (TaKaRa, Dalian, China), and 20 ng of genomic DNA template. PCR was performed on a thermocycler (MJ Research Inc., Bio-Rad, USA) under the following conditions: 95°C for 5 min; 30 cycles of 94°C for 40 s, 50°C for 40 s, and 72°C for 1 min; and 72°C for 5 min. The PCR products were purified using TaKaRa Agarose Gel DNA Purification Kit (TaKaRa, Dalian, China) and quantified with NanoDrop. A mixture of PCR products was prepared by mixing 200 ng of the purified 16S amplicons from each sample, and then pyrosequenced on ROCHE 454 FLX Titanium platform.

### Calculation of species richness and taxonomic assignment of pyrosequencing reads

The pyrosequencing data were deposited in NCBI Sequence Read Archive (SRA) database with accession number SRA065792. The downstream bioinformatics analysis was performed using QIIME^38^. Raw reads with an average flowgram score of 25 or less over a window of 50 bp, shorter than 150 bp, with one or more ambiguous nucleotides, containing homopolymers of more than 6 bp or without a complete barcode were excluded from subsequent analysis. The remaining reads were assigned to corresponding samples according to their barcodes and then subjected to flowgram correction using Denoiser^39^. Denoised reads were clustered using UCLUST and then assigned to OTUs at 97% similarity. The most abundant reads of each OTU were selected as representatives and aligned using PyNAST^40^ against the Silva108 database^41^. Chimeric reads were identified with ChimeraSlayer^42^ and removed. Rarefaction curves, alpha diversity, and beta diversity were computed as parts of QIIME pipeline. To assess consistency of microbial communities between the replicates, the percentages of phyla and genera in the replicates were compared individually using Mann-Whitney test.

### Similarities and correlations of microbial communities

Similarities between microbial communities from different sources (BP-biofilm, SV-biofilm and SW) were determined by phylogenetic distance among reads using UniFrac^43^ and jackknifed UPGMA clustering implemented on the QIIME beta diversity pipeline. Taxonomic assignment of qualified reads was performed by RDP classifier^44^ against Silva108 with a bootstrap confidence of 50%. The relative abundance of reads assigned to different taxa served as the input for generating a heatmap using Cluster3^45^. Taxa with small differences in relative abundance among samples were filtered out by applying a threshold divergence of 0.5%. The remaining taxa were then normalized and centered by mean. A hierarchical cluster was generated by using the complete linkage method with a metric of correlation (uncentered).

Percentage abundance data of the microbial groups at phylum and genus levels in each library were used as ‘species data’ and the environmental variables (site, type of material and plate position) as ‘environmental data’. The length of gradient for the species data was first checked with the detrended correspondence analysis (DCA) to determine which model would be most suitable for correlation analysis. Since results of DCA indicate a linear model at the phylum level, the redundancy analysis (RDA) was used for direct gradient analysis and canonical correspondence analysis (CCA) was used for a unimodal model at the genus level. Both RDA and CCA aimed to determine the extent of variations associated with the factors that contribute individually or in combination. Therefore, ordination methods using CANOCO (version 4.5, Microcomputer Power, USA) were used to correlate the microbial assemblages with site, type of material, and plate position. Tests of significance were performed for all canonical axes with 499 Monte-Carlo permutations under a reduced model. Automatic forward selection was applied to build the optimal models of microbe-environment relationship, where appropriate.

### Clone library construction

A clone library was constructed for the *dsrB* gene using the primer pairs dsrp2060F^46^ and dsr4R^47^, in 20 μL mixtures of 1.25 U of Taq DNA polymerase (New England BioLabs, England), 2 μL of 10× PCR buffer, 1 μL of dNTPs (2.5 mM), 0.5 μL of each primer (10 μM) and ~30 ng of DNA template. PCR products were purified using the NucleoSpin® Gel and PCR Clean-up kit (Macherey-Nagel, France) and cloned into vectors using the TOPO TA cloning kit (Invitrogen, USA) according to the manufacturer's instructions. A total of 96 positive clones were obtained after screening 100 clones of the *dsrB* library. Sequencing was performed on a DNA analyzer (ABI 3730, Applied Biosystems). Representative sequences of each OTU, assigned at 3% dissimilarity level, were aligned with reference sequences from the GenBank and phylogenetic trees were constructed by the neighbor-joining method using the software package Mega 5.1. The clone sequences were deposited in the GenBank under the accession number KC857513-KC857543.

## Supplementary Material

Supplementary Information supplementary file

## Figures and Tables

**Table 1:** Environmental parameters of brine from Thuwal Seeps. Values for salinity, temperature, pH, dissolved oxygen, and methane concentration are measured by the CTD unit and are averages ± SD of 30 continuous real-time measurements. Those for sulfate, manganese, iron, copper, silica, nitrate, nitrite and phosphorus are from HACH system (n = 3), while that for cell density is based on DAPI count (n = 3)

Measurement	Seeping water
Salinity (ppt)	116.71 ± 2.44
Temperature (°C)	28.76 ± 1.72
pH	6.82 ± 0.33
Dissolved oxygen (%)	17.17 ± 4.97
Methane (μM)	0.30 ± 0.00
Sulfate (g/L)	2.59 ± 0.01
Manganese (mg/L)	0.79 ± 0.03
Iron (mg/L)	0.00 ± 0.00
Copper (μg/L)	14.83 ± 0.32
Silica (mg/L)	10.60 ± 0.36
Nitrate (mg/L)	1.32 ± 0.08
Nitrite (μg/L)	6.26 ± 0.06
Phosphorus (mg/L)	0.24 ± 0.02
Cell density (×10^3^ cells/mL)	95.99 ± 20.24

**Table 2:** Characteristics of biofilms. The BP-biofilms were developed within the brine pool for 72 h, and the SV-biofilms were developed at the seep vent for 100 h. Biofilms were visualized by confocal laser scanning electron microscopy and z-stack images were analyzed by the software PHYLIP. Values are mean ± SD of 3 images. Polystyrene plates from the seep vent were lost during sample processing and thus no value was recorded

		BP-biofilms		SV-biofilms	
	Material	Inside	Outside	Inside	Outside
Cell density (× 10^3^ cell/cm^2^)	PS	8.35 ± 2.39	15.99 ± 3.62	/	/
	PVC	12.01 ± 1.52	16.90 ± 5.54	23.61 ± 6.50	24.73 ± 9.99
Biovolume (× 10^3^ μm^3^)	PS	549.28 ± 105	596.42 ± 407	/	/
	PVC	863.98 ± 155	864.74 ± 383	1007.27 ± 792	1024.30 ± 184
Mean thickness (μm)	PS	15.08 ± 5.15	17.13 ± 8.93	/	/
	PVC	15.69 ± 2.89	25.79 ± 10.28	17.51 ± 7.34	40.13 ± 17.71
Roughness	PS	0.47 ± 0.04	0.44 ± 0.05	/	/
	PVC	0.48 ± 0.01	0.46 ± 0.05	0.48 ± 0.01	0.47 ± 0.02
Coverage (%)	PS	53.75 ± 7.08	66.54 ± 9.50	/	/
	PVC	54.88 ± 4.02	57.03 ± 6.48	52.26 ± 2.93	50.07 ± 1.82

**Table 3:** Diversity of microbial communities of seeping water and biofilms. The biofilms were developed within the brine pool and on a seep vent at Thuwal Seeps. Materials of substrata (Al: Aluminum; PS: Polystyrene; PVC: polyvinyl chloride) and positions on the mounting platforms (outside and inside) were different between biofilm samples. The microbial communities were revealed based on analysis of pyrosequenced 16S rRNA amplicons. Number of OTUs was calculated at 97% similarity level based on the entire qualified reads (the number is shown). Shannon index was based on datasets normalized to the smallest library size (i.e. 471 reads)

		Site						
Sample ID	Type	Location	Duration	Material	Position	No. of reads	No. of OTUs	Shannon index
BA	Biofilm	Pool	72 h	Al	Outside	5201	412	5.97
BAI	Biofilm	Pool	72 h	Al	Inside	9120	420	6.80
BS	Biofilm	Pool	72 h	PS	Outside	12831	330	5.95
BSI	Biofilm	Pool	72 h	PS	Inside	12357	316	5.93
BC	Biofilm	Pool	72 h	PVC	Outside	471	39	4.39
BCI	Biofilm	Pool	72 h	PVC	Inside	1275	71	3.75
SA	Biofilm	Vent	100 h	Al	Outside	10807	109	1.85
SAI	Biofilm	Vent	100 h	Al	Inside	8748	38	1.67
SS	Biofilm	Vent	100 h	PS	Outside	7328	91	2.11
SSI	Biofilm	Vent	100 h	PS	Inside	24899	138	1.41
SC	Biofilm	Vent	100 h	PVC	Outside	17543	239	2.33
SCI	Biofilm	Vent	100 h	PVC	Inside	1804	44	3.20
SW	Water	Vent	/	/	/	7001	400	6.44

**Table 4:** Summary of ordination analysis. RDA and CCA were performed for each and all of the 3 factors (site, type of substrate material, attachment position on the platform) to determine the explained variance in microbial community composition at the phylum and genus levels, respectively. Only the first and second axes were shown. P-value was calculated based on Monte Carlo test for all canonical axes

Phylum level								
	Site only		Material only		Position only		All	
Axes	1	2	1	2	1	2	1	2
Eigenvalues	0.778	0.140	0.035	0.006	0.008	0.907	0.800	0.018
Cumulative percentage variance								
of species data	77.8	91.8	3.5	4.0	0.8	91.5	80.0	81.8
of species-environment relations	100.0	0.0	85.5	100.0	100.0	0.0	96.8	98.8
Sum of all canonical eigenvalues	0.778		0.040		0.008		0.827	
P-value	0.004		0.884		0.902		0.010	

Genus level								
	Site only		Material only		Position only		All	
Axes	1	2	1	2	1	2	1	2
Eigenvalues	0.903	0.676	0.266	0.153	0.174	0.907	0.905	0.289
Cumulative percentage variance								
of species data	31.8	55.5	9.4	14.8	6.1	38.1	31.8	42.0
of species-environment relations	100.0	0.0	63.5	100.0	100.0	0.0	60.5	79.8
Sum of all canonical eigenvalues	0.903		0.420		0.174		1.497	
P-value	0.004		0.820		0.924		0.004	
