# Supplementary material for: In situ environment rather than substrate type dictates microbial community structure of biofilms in a cold seep system
Source: Sci Rep. 2014 Jan 8;4:3587. doi: 10.1038/srep03587 (PMC5378041; doi:10.1038/srep03587)
Supplement: Supplementary Information — supplementary file [file srep03587-s1.pdf]

In situ environment rather than substrate type dictates microbial community structure of biofilms in a cold seep system

Authors

On On Lee, Yong Wang, Renmao Tian, Weipeng Zhang, Chun Shum Shek, Salim Bougouffa, Abdulaziz Al-Suwailem, Zenon B. Batang, Wei Xu, Guang Chao Wang, Xixiang Zhang, Feras F. Lafi, Vladmir B. Bajic, Pei-Yuan Qian

Table S1 Diversity of microbial communities of replicated seeping water and biofilms developed on different substrata for different durations and positions on the mounting platforms at Thuwal Seeps based on 16s rRNA gene pyrosequencing. The number of OTUs was calculated at 97% similarity level based on the entire dataset. The 8-nucleotide barcode, unique to individual samples and added to the primers, enables multiplexing different biofilm samples in a single pyrosequencing run. Observed species and Shannon index were based on datasets normalized to 551 reads per sample.

Sample ID	Barcode	No. of reads	No. of OTUs	Observed species	Shannon
BA	ATGACGAC	1734	320	160	6.32
BAI	TGCTCTCA	282	63	-	-
BS	CTCATCGA	23590	926	193	6.74
BSI	TCAGTGCA	1649	233	123	5.72
BC	GCTAGCTA	551	132	132	5.97
BCI	ATATGCGC	2177	100	56	3.28
SA	TACGAGAC	15925	211	31	2.10
SAI	TAGCGACA	17181	99	18	1.78
SS	GTCGCGTA	5686	197	44	2.87
SSI	TATCTCGC	9293	288	66	3.53
SC	TCGTATGC	3353	123	59	3.58
SCI	CGAGTCTC	29950	272	26	1.74
SW	TCAGTGCA	2046	230	136	6.44
		113387			

Fig. S1. SEM micrographs of clumps observed exclusively in the biofilms developed on PVC plates. These structures were more frequently encountered in the SV-biofilms and taller in the biofilms developed on plates glued to the outer side of the aluminum platform.

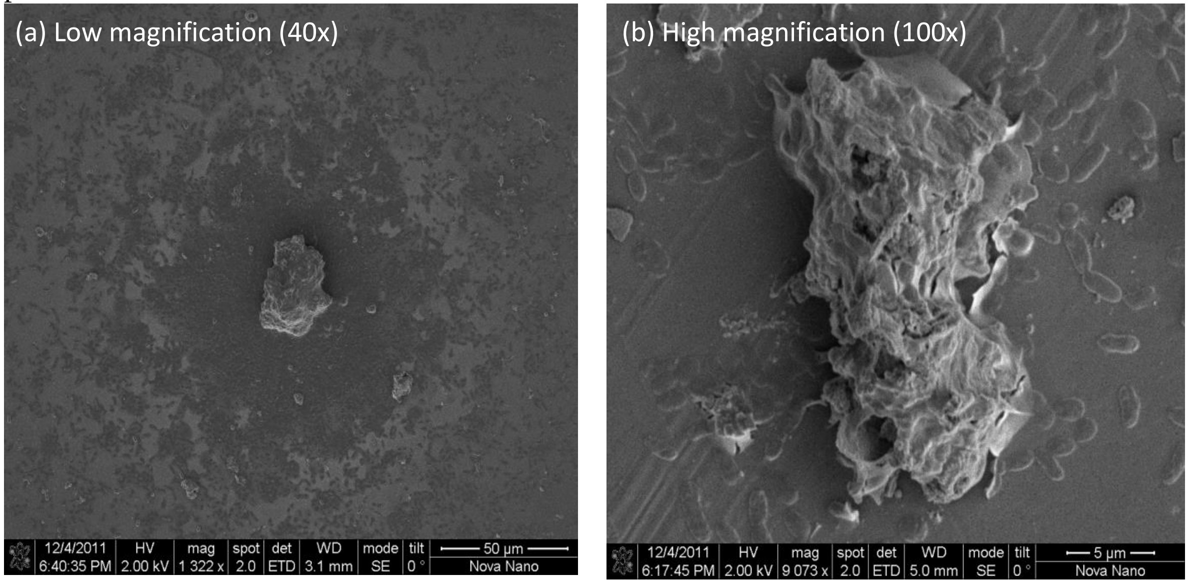

Fig. S2. Relative abundance (%) of microbial taxa classified based on 16S rRNA gene sequences from replicated samples of seeping water and biofilms developed on different substrate materials for different durations at the Thuwal Seeps. The BP-biofilms were developed within the brine pool for 72h while the SV-biofilms were developed at the seep vent for 100h. Sequences were obtained by pyrosequencing and classified by comparing with the SILVA database at (a) phylum and (b) genus levels. Minor group represents the sum of all phyla or genera with relative abundances of less than 1% and 3%, respectively, for all 13 samples. Refer to Table 3 for sample ID.

(a)

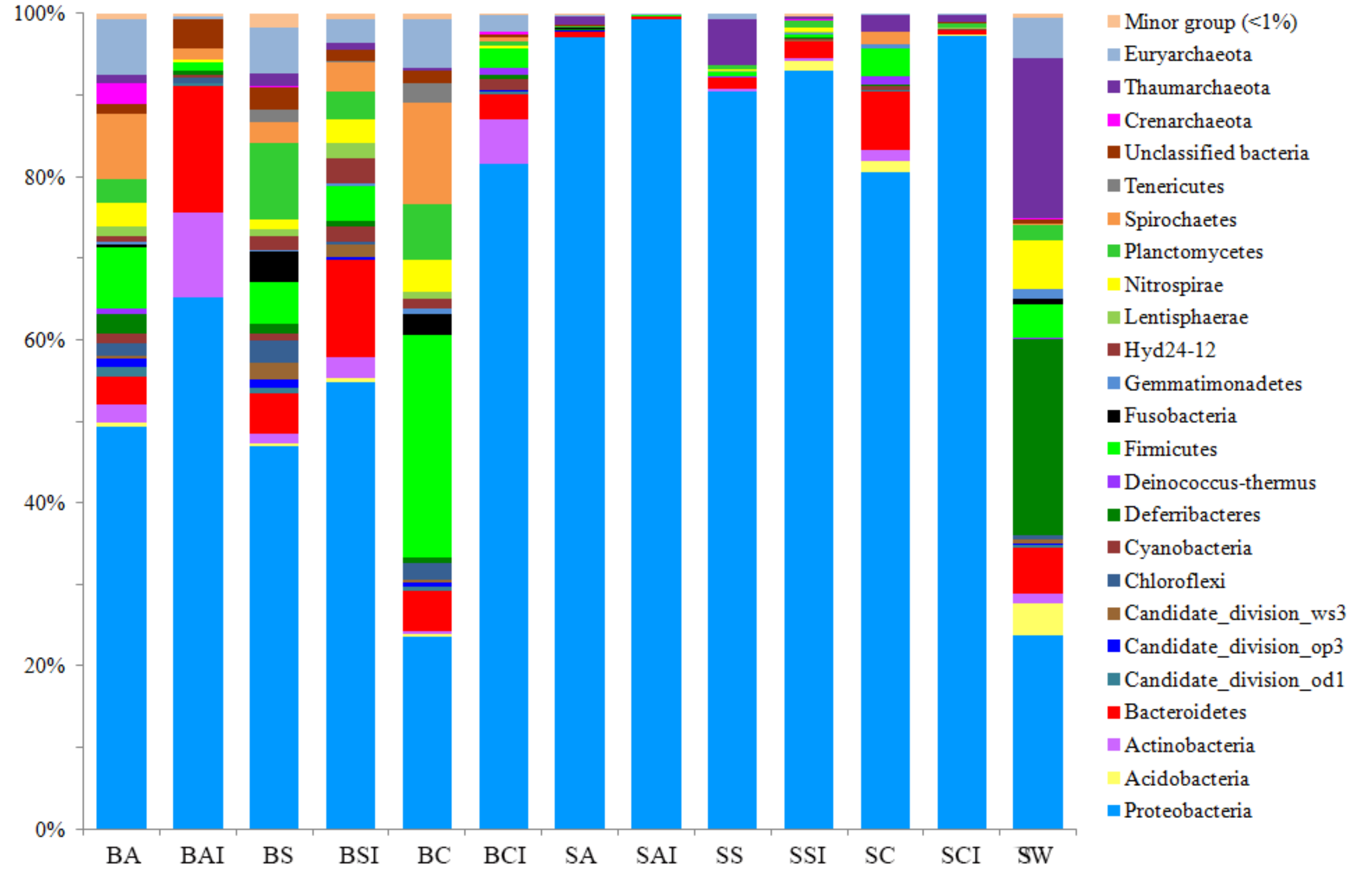

(b)

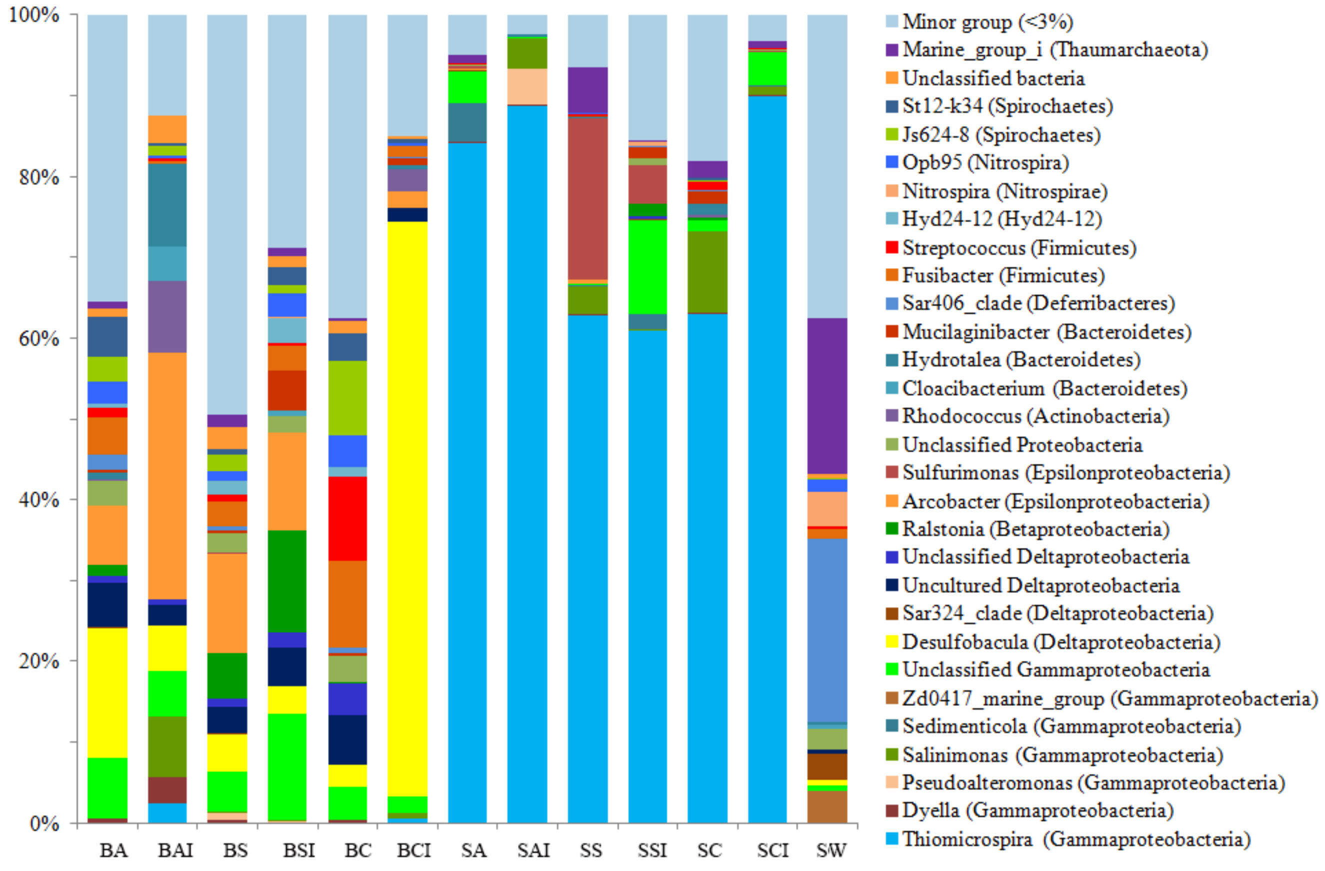
100%
80%
60%
40%
20%
0%
BA
BAI
BS
BSI
BC
BCI
SA
SAI
SS
SSI
SC
SCI
SW
Minor group (<3%)
Marine_group_i (Thaumarchaeota)
Unclassified bacteria
St12-k34 (Spirochaetes)
Js624-8 (Spirochaetes)
Opb95 (Nitrospira)
Nitrospira (Nitrospirae)
Hyd24-12 (Hyd24-12)
Streptococcus (Firmicutes)
Fusibacter (Firmicutes)
Sar406_clade (Deferribacteres)
Mucilaginibacter (Bacteroidetes)
Hydrotalea (Bacteroidetes)
Cloacibacterium (Bacteroidetes)
Rhodococcus (Actinobacteria)
Unclassified Proteobacteria
Sulfurimonas (Epsilonproteobacteria)
Arcobacter (Epsilonproteobacteria)
Ralstonia (Betaproteobacteria)
Unclassified Deltaproteobacteria
Uncultured Deltaproteobacteria
Sar324_clade (Deltaproteobacteria)
Desulfobacula (Deltaproteobacteria)
Unclassified Gammaproteobacteria
Zd0417_marine_group (Gammaproteobacteria)
Sedimenticola (Gammaproteobacteria)
Salinimonas (Gammaproteobacteria)
Pseudoalteromonas (Gammaproteobacteria)
Dyella (Gammaproteobacteria)
Thiomicrospira (Gammaproteobacteria)

Fig. S3. Similarity of microbial communities of replicated samples of seeping water and biofilms developed on different substrate materials for different durations at the Thuwal Seeps. Data were based on 16S rRNA gene pyrosequencing and similarity was calculated based on UniFrac analysis and displayed as UPGMA jackknifed cluster. Bootstrap values of 1,000 replications are shown at nodes. Scale bar indicates 5% divergence among the microbial communities. Refer to Table 2 for sample ID.

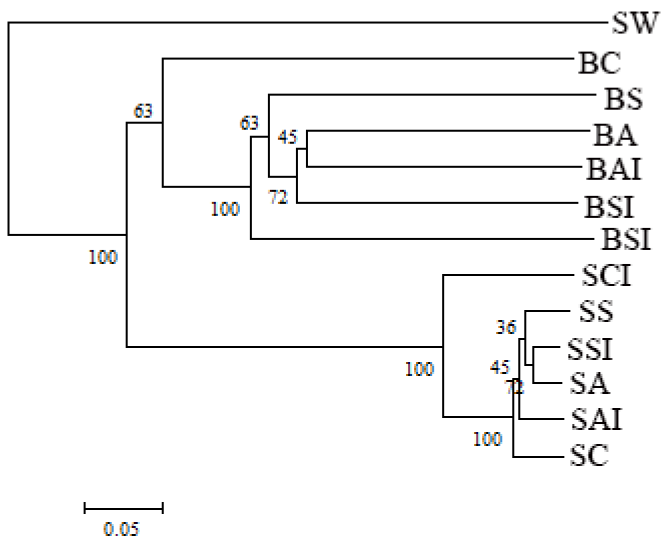

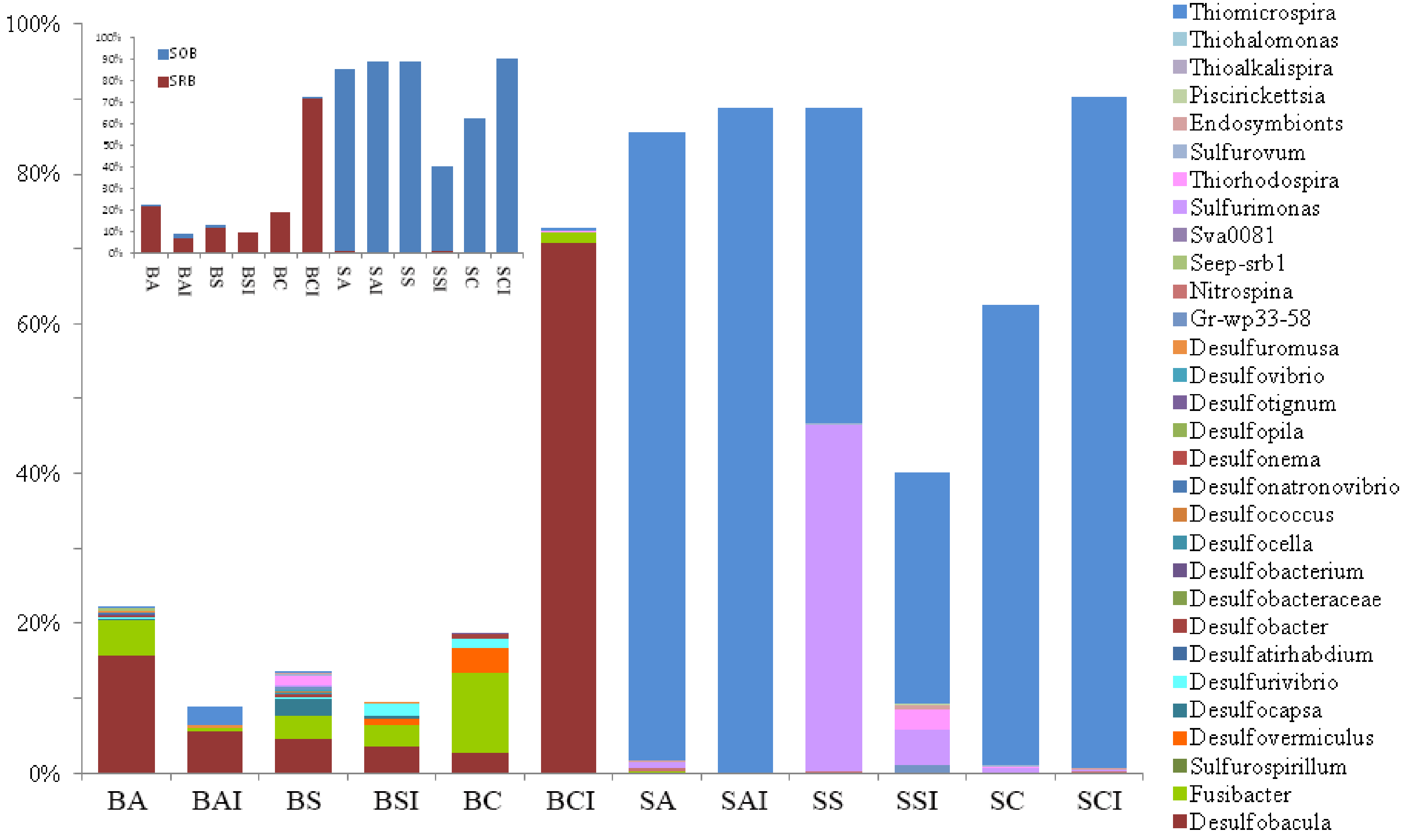

Fig. S4 Relative abundance of 16S rRNA gene pyrosequencing reads from the replicated samples of biofilms and seeping water assigned to different groups of sulfate-reducing (SRB) and sulfur-oxidizing (SOB) bacteria in different biofilms. Inset shows the relative abundance of total SRB and SOB. Taxonomic classification was based on comparison of sequences with the SILVA database. Refer to Table 3 for sample ID.
